# Early-stage hypopharyngeal squamous cell carcinoma treated with radical radiotherapy at a uniform dose of 70 Gy in 35 fractions: a single-center study

**DOI:** 10.1007/s00405-024-08722-w

**Published:** 2024-05-08

**Authors:** Atsuto Katano, Hideomi Yamashita

**Affiliations:** Department of Radiology, The University of Tokyo Hospital, 7-3-1 Hongo, Bunkyo-ku, Tokyo, 113-8655 Japan

**Keywords:** Hypopharyngeal carcinoma, Head and neck neoplasms, Early stage, Radical radiotherapy, Squamous cell carcinoma of head and neck

## Abstract

**Introduction:**

Hypopharyngeal squamous cell carcinoma (HSCC) is often undetected until advanced stages, which contributes to poor survival rates. Recent advances in diagnostic techniques have enhanced the feasibility of early detection, and this study evaluated the efficacy and safety of radical radiotherapy that specifically targets early stage HSCC.

**Methods:**

This retrospective cohort study consecutively analyzed patients with clinical stage I or II HSCC between December 2008 and February 2023. These patients underwent radical radiotherapy with a uniform dose of 70 Gy delivered in 35 fractions to the primary site, followed by elective nodal irradiation. We assessed clinical outcomes, including overall survival (OS), disease-free survival (DFS), and 5-year locoregional control (LRC). Multivariate analyses were performed to identify the independent prognostic factors for OS.

**Results:**

The 5-year OS rate of the entire cohort was 80.7% (95% confidence interval [CI] = 66.5–89.4%), with no significant difference between patients with clinical stage I and II HSCC. Stratified by subsite, the 5-year OS for pyriform sinus, posterior pharyngeal wall, and postcricoid region were 81.6, 68.2, and 100%, respectively. The ECOG-Performance status (PS) was identified as an independent risk factor for OS (hazard ratio [HR] = 8.457; 95% CI 1.325–53.970; p = 0.024). DFS at 5 years was 66.4%, with local recurrence being the most frequent, and LRC rate at 5 years was 79.3%. Acute and late-phase toxicities were predominantly mild to moderate, with no grade 3 or higher toxicities reported.

**Conclusion:**

This study supports radical radiotherapy as an effective approach for optimal tumor control in patients with early stage HSCC. Despite the limitations of this study, including its retrospective design and single-center confinement, our results revealed the effectiveness and feasibility of radical radiotherapy in the management of early stage HSCC.

## Introduction

Hypopharyngeal squamous cell carcinoma (HSCC) often escapes early detection due to its subtle symptoms and deep-seated location. More than 50% of patients with hypopharyngeal cancers present at an advanced stage, and HSCC is associated with the lowest 5-year relative survival rate among head and neck cancers [1–3]. Fortunately, early detection of hypopharyngeal carcinoma has recently become more feasible, and narrow band imaging (NBI) has emerged as a promising endoscopic technique in the diagnosis of head and neck cancers [4, 5]. Moreover, recent advancements in artificial intelligence-assisted NBI methods have significantly enhanced the detection of early stage cancers by employing new diagnostic approaches in oncology [6].

Traditionally, operable hypopharyngeal cancer has been treated with total laryngectomy or circumferential pharyngectomy [7]. Radiotherapy has emerged as a crucial therapeutic modality for head and neck cancers that offers a noninvasive approach with the potential for organ preservation [8]. The National Cancer Database, analyzed by Burbure et al., includes 9314 patients diagnosed with hypopharynx cancer between 2004 and 2016, and the database results have revealed that treatment modality does not influence the survival of patients with early stage HSCC [9]. However, the clinical outcomes of radiotherapy for early stage hypopharyngeal carcinoma are currently limited and require further research. Our study focused on evaluating the efficacy and safety of radical radiotherapy that delivers a uniform dose of 70 Gy in 35 fractions, to specifically target the primary site of early stage HSCC through elective nodal irradiation.

## Methods

This retrospective cohort study assessed clinical outcomes following radical radiotherapy in individuals diagnosed with early stage HSCC. The cohort comprised consecutively enrolled patients with a confirmed pathological diagnosis of squamous cell carcinoma of the hypopharynx, clinically staged as I or II. Each patient underwent radical radiotherapy at our institution between December 2008 and February 2023 and received a consistent dose of 70 Gy, delivered in 35 fractions to the primary site. Patients with a history of hypopharyngeal carcinoma or previous irradiation of the head and neck region were excluded. We also excluded patients who were lost to follow-up prematurely, because their absence hindered the comprehensive evaluation of clinical outcomes. The study was conducted in accordance with the ethical standards outlined in the Declaration of Helsinki. Ethical approval was obtained from the Institutional Review Board of the University of Tokyo Hospital. Patient confidentiality and anonymity were maintained throughout this study.

### Radiotherapy

Hypopharyngeal tumors are distinguished by their propensity for local invasion and lymphatic dissemination [10]. All study patients were treated by radiotherapy targeting the primary site, with 70 Gy in 35 fractions by elective nodal irradiation (ENI). ENI was administered using either a 2-step or a simultaneous integrated boost (SIB) method. All patients underwent three-dimensional conformal radiation therapy (3D-CRT) or intensity radiation therapy (IMRT), including helical tomotherapy. The clinical target volume (CTV) for the primary tumor and ENI was delineated according to international guidelines [11, 12].

For radiotherapy administration, a thermoplastic mask was placed on the patient in the supine position for immobilization. CT scans were performed from the top of the skull to the bifurcation of the trachea level in 2-mm thick slices. Gross tumor volume (GTV) was determined using CT, MRI, or PET/CT visualization. Considering the anatomical structure, the CTV was defined as the GTV with a 10 mm margin, at least in the craniocaudal direction. The planning target volume (PTV) was defined as the CTV plus a 3–5 mm margin. The prescribed dose to the PTV was 70 Gy, delivered in 2 Gy fractions over the course of 35 fractions. For ENI, the prescribed dose ranged from 40 to 46 Gy over 20–23 fractions using the two-step method, or 54 Gy over 35 fractions using the SIB method.

**Fig. 1:**
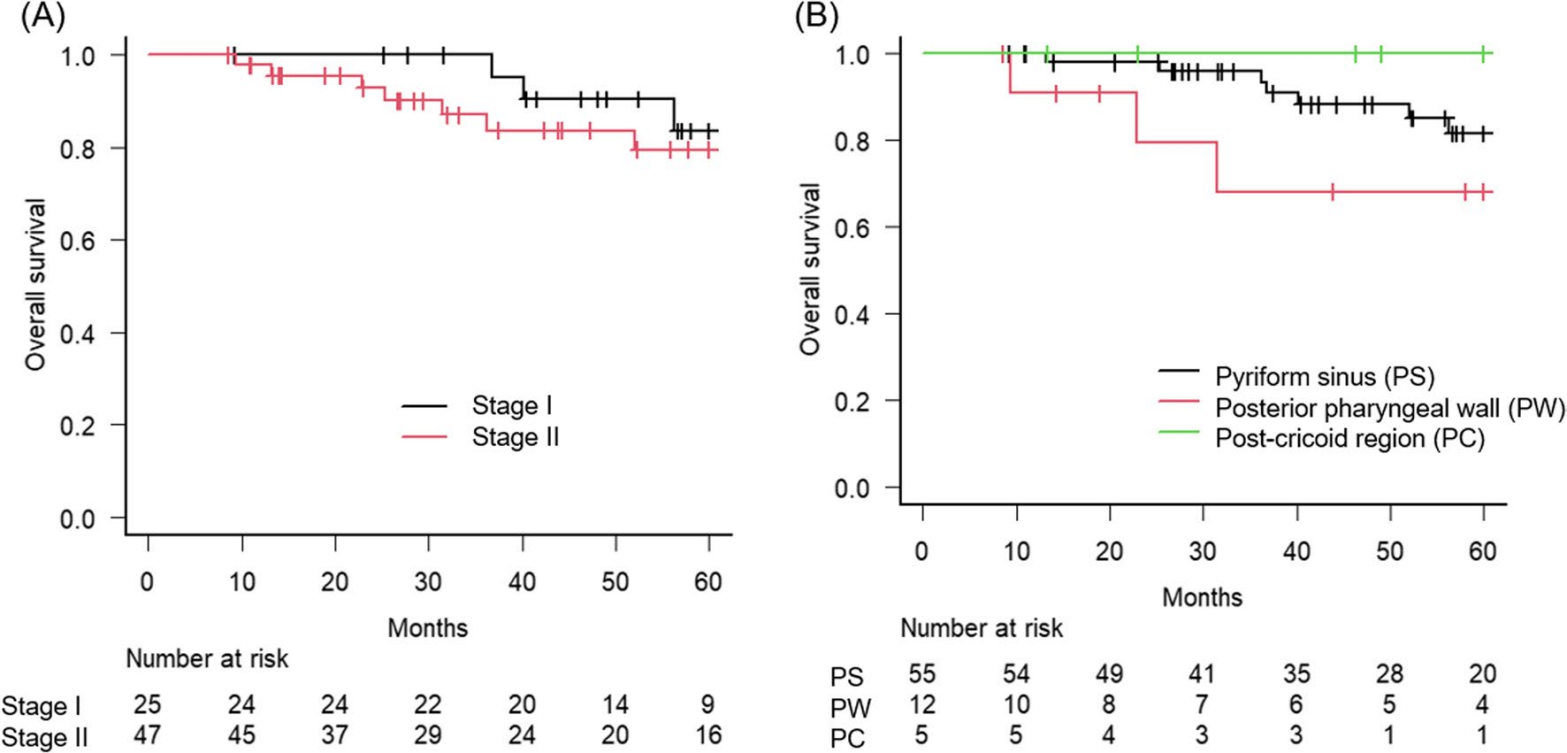
Kaplan–Meier curves of the overall survival, stratified by **A** clinical stage and **B** tumor subsite (pyriform sinus, posterior pharyngeal wall, and post-cricoid region)

The clinical outcomes that were assessed included OS, disease-free survival (DFS), and locoregional control (LRC) rates at 5 years. OS was defined as the time from the initiation of radiotherapy to death from any cause. DFS was defined as the time from radiotherapy initiation to disease progression or death from any cause, whichever occurred first. LRC was defined as the absence of a local or regional recurrence. Additionally, specific recurrence sites and salvage treatments have been documented in patients who experience disease recurrence. The laryngeal preservation survival rate (LPS) was defined as the time from the initiation of radiotherapy to pharyngolaryngectomy or death from any cause, whichever occurred first (Fig. 1).

**Fig. 2:**
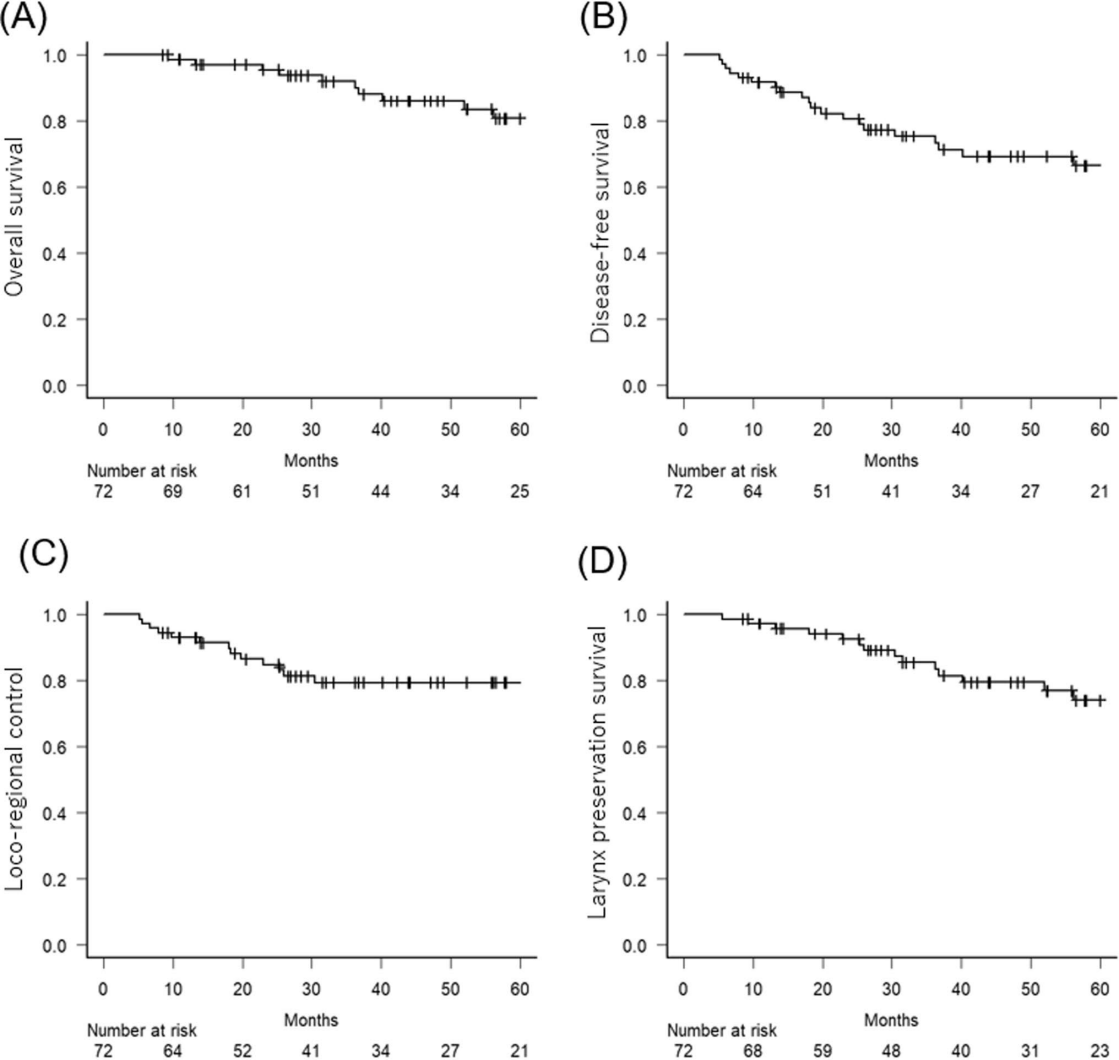
Kaplan–Meier curves for entire cohort of overall survival (**A**), disease-free survival (**B**), and loco-regional control (**C**), and larynx preservation survival rates (**D**)

Survival rates were estimated using the Kaplan–Meier method and 95% CIs were calculated. The log-rank test was used to compare survival curves between groups. Univariate and multivariate analyses were conducted using Cox proportional hazards regression to identify independent prognostic factors for OS. All statistical analyses were performed using R software, and differences with p-values less than 0.05 were considered statistically significant (Fig. 2).

## Results

This study included 72 patients with a median age of 71 years (range, 41–89 years), and the median follow-up period was 48.7 months (range: 8.5–161 months). Table 1 summarizes patient and treatment characteristics. The majority of patients (93%) had a favorable ECOG-PS of 0. The distribution of the tumor subsites included 55 in the pyriform sinus, 12 in the posterior pharyngeal wall, and 5 in the post-cricoid region. Clinical staging revealed stage I and stage II HSCC in 25 and 47 patients, respectively. In terms of sex, 67 patients were male and five were female. Seventeen patients received chemotherapy with radical radiotherapy, all of whom were classified as clinical stage II, and the vast majority (15 patients) had a good performance status, with an Eastern Cooperative Oncology Group-Performance Status (ECOG-PS) of zero. Additionally, among these patients, six received induction chemotherapy, 10 received concomitant chemotherapy, and one received both modalities. All induction chemotherapy regimens consisted of docetaxel plus 5-fluorouracil and cisplatin (DCF), and the concomitant chemotherapy was typically a triweekly cisplatin regimen. Most patients in this cohort (n = 56) had other synchronous or metachronous cancers, with esophageal cancer being the most common (n = 35).

**Table 1:** Characteristics of 72 patients with early-stage hypopharyngeal squamous cell carcinoma treated by radical radiotherapy

Variable	Number (percentage)
Age: median [range]	71 [41–89]
Sex	
Male	67 (93%)
Female	5 (7%)
ECOG-performance status	
0	67 (93%)
1	5 (7%)
Tumor subsite	
Pyriform sinus	55 (76%)
Posterior pharyngeal wall	12 (17%)
Post-cricoid region	5 (7%)
Clinical stage	
I	25 (35%)
II	47 (65%)
Chemotherapy	
No	55 (76%)
Induction	6 (8%)
Concomitant	10 (14%)
Induction and concomitant	1 (1%)
Synchronous or metachronous cancers	
No	16 (22%)
Yes	56 (78%)

We assessed clinical outcomes following radical radiotherapy. The OS rate at 5 years for entire cohort was 80.7% (95% CI 66.5–89.4%), indicating that 14 patients had died during the analysis period. The 5-year OS for clinical stages I and II were 83.5% (95% CI 56.2–94.5) and 79.5 (95% CI 60.9–89.9%), respectively. Patients with stage I disease had a slightly higher OS rate than those with stage II disease; however, this difference was not statistically significant (log-rank test, p = 0.309). The 5-year OS rate was stratified by subsite, i.e., pyriform sinus, posterior pharyngeal wall, and post-cricoid region were 81.6% (95% CI 64.6–91.0%), 68.2% (95% CI 29.7–88.6%) and 100% (95%CI: not assessed), respectively.

The leading cause of death in the cohort was other cancer types (seven patients), followed by hypopharyngeal cancer (five patients), pneumonia (one patient), and accidents (one patient). The hypopharyngeal cancer specific survival rate at 5 years for entire cohort was 90.6% (95% CI 78.3–96.1%).

The DFS rate at 5 years was 66.4% (95% CI 52.3–77.2%), indicating that 19 patients experienced disease progression during this period. The most frequent recurrence site was local in 14 patients, followed by regional lymph nodes in two, lungs in one, liver in one, and both lungs and regional lymph nodes in one. The LRC rate at 5 years was 79.3% (95% CI 66.9–87.5%).

Among the three patients who experienced recurrence with distant metastasis, two had received systemic therapy with DCF, and one chose palliative care (BSC). Both patients with regional lymph node recurrence were successfully treated with neck dissection. Of the 14 patients who experienced local recurrence, five were salvaged by total pharyngolaryngectomy (TPL), four underwent partial resection, including ESD without TPL, four underwent BSC, and one underwent systemic pembrolizumab. The LPS rate at 5 years was 74.0% (95% CI 59.4–84.0%).

The results of the univariate and multivariate Cox proportional hazards models that evaluated the independent potential prognostic value of various parameters for OS are shown in Table 1. Univariate analyses revealed no significant differences in any of the parameters. In multivariate analysis, ECOG-PS was identified as an independent risk factor (HR = 8.457; 95% CI 1.325–53.970; p = 0.024).

The predominant acute-phase toxicities during radical radiotherapy are grades 1–2 mucositis, odynophagia, and dermatitis. Most patients experience mild to moderate toxicities. Conversely, during the late phase, the prevalent toxicities included grade 1–2 xerostomia, observed in 47 patients (91%) and dysphagia, noted in 29 patients (55%). No radiation-induced toxicities exceeding grade 3 were reported in either the acute or the late phases of radical radiotherapy (Table 2).

**Table 2:** Univariate and multivariate Cox proportional hazard analysis of overall survival for a cohort of 72 patients

Covariable		Univariate		Multivariate	
		Hazard ratio [95% CI]	p value	Hazard ratio [95% CI]	p value
Age	≤ 70 vs. > 70 years	1.369 [0.476–3.935]	0.560	1.036 [0.280–3.828]	0.958
Sex	Male vs. Female	1.791 [0.231–13.900]	0.577	0.817 [0.072–9.297]	0.871
ECOG-Performance status	0 vs. 1	4.751 [0.958–23.550]	0.056	8.457 [1.325–53.970]	0.024
Tumor subsite	Pyriform sinus vs. others	2.813 [0.912–8.672]	0.072	3.135 [0.837–11.740]	0.090
Clinical stage	I vs. II	1.828 [0.572–5.848]	0.309	2.114 [0.539–8.296]	0.283
Chemotherapy	No vs. Yes	0.854 [0.236–3.095]	0.810	0.663 [0.155–2.829]	0.579
Other cancers	No vs. Yes	0.673 [0.181–2.498]	0.554	0.597 [0.125–2.855]	0.518

## Discussion

The findings of this study support the idea that radical radiotherapy offers an effective approach to achieve optimal tumor control while carefully managing the risk of radiation-related toxicity.

In a study by Sato et al., a cohort of 33 patients, including 12 with stage I and 21 with stage II HSCC, underwent radical radiotherapy for early-stage disease [13]. Their study, with a median follow-up period of 81 months, reported a 5-year OS rate of 58% and an LRC rate of 56%. Nishimura et al. investigated the treatment outcomes of early stage HSCC in 45 patients who underwent radiotherapy, including 27 with stage I and 18 with stage II HSCC, and reported favorable results with a median follow-up period of 62 months [14]. The 5-year OS rate was 81%, and the 5-year LRC rate reached 83%. Nakamura et al. conducted a multi-institutional analysis of 115 patients with early hypopharyngeal cancer who were treated with radical radiotherapy across 10 institutions [15]. Among them, 39 had stage I disease and 76 had stage II disease, with a median age of 67 years. The 5-year survival rates were 66.0% overall and 77.4% were disease-specific survival in these patients.

Primary transoral surgery (TORS) is considered the cornerstone treatment for patients with early stage HSCC who exhibit good general health and adequate daily functions [16]. A French collaborative multicenter study investigated the clinical outcomes of TORS in 57 patients diagnosed with early stage pyriform sinus carcinoma [17]. The study reported favorable results, with a 2-year overall survival and disease-free survival rate of 84% and 74%, respectively, after a median follow-up of 23 months. Additionally, the study also revealed an excellent organ preservation rate of 96%. Furthermore, Casanueva et al. assessed 34 patients with early stage HSCC treated with transoral laser microsurgery. The findings revealed a 5-year OS rate of 51%, a disease-specific survival rate of 66%, and a 5-year local control rate of 92% [18].

The leading cause of death in our study was from other cancers, indicating the importance of continued surveillance for synchronous or metachronous malignancies, especially considering that 56 of our 72 patients had additional cancers, with esophageal cancer being the most common. Patients with head and neck cancer exhibit an elevated likelihood of developing simultaneous or subsequent second primary cancers that originate from the upper aerodigestive tract, lungs, and esophagus [19, 20]. Tseng et al. reported that after primary hypopharyngeal cancer treatment, patients are at risk of continuous development of metachronous esophageal cancer up to a decade later [21]. The authors insisted that long-term endoscopic surveillance may be necessary for these patients.

The identification of therapeutic biomarkers could improve treatment strategies by offering a more tailored and effective therapeutic approach for hypopharyngeal cancer. Sato et al. assessed the prognostic value of pretreatment fibrinogen-to-lymphocyte ratio (FLR) in 95 patients who received definitive radiotherapy for HSCC and concluded that a high pretreatment FLR was an independent prognostic factor for poor PFS (HR = 2.14, p = 0.026) and OS (HR = 2.86, p = 0.024) [22]. Furthermore, Ishinaga et al. investigated the role of circulating miR-21 as a predictive biomarker in head and neck squamous cell carcinoma patients undergoing chemoradiotherapy[23]. Plasma miR-21 levels were higher in patients, particularly in those with recurrence and poor OS. AI models for predicting cancer control and toxicity outcomes in head and neck cancer RT have shown promising performance and may be highly useful for individualized risk-based decision-making. Notably, Bang et al. proposed that AI models have substantial clinical utility, offering the potential for individualized, risk-based decision making in the treatment of patients with head and neck cancers [24]. The findings of our study revealed that performance status is an independent risk factor for patient prognosis. Performance status has been found to play a crucial role in cancer treatment and is considered a predictor of treatment response in patients with head and neck cancer patients [25, 26]. Additionally, this might indicate that assessing the performance status is more important than a patient’s actual age when considering prognosis in the context of early HSCC management.

The strength of our study lies in the consistent administration of uniform doses in all patients. Nevertheless, this study has some limitations. First, treatment heterogeneity is associated with the use of both 3D-CRT and IMRT. Second, the retrospective design of this study might have introduced selection bias and confounding factors. Third, confinement to a single center hampered the generalizability of our findings. Finally, the sample size may have been insufficient to identify rare adverse events.

## Conclusion

In summary, our analyses revealed favorable clinical outcomes for LRC and OS among patients undergoing definitive radiotherapy for early stage HSCC. Hypopharyngeal cancer is often diagnosed at an advanced stage, leading to a poor prognosis. However, early detection can facilitate the implementation of the highly effective treatment interventions presented in this study.

## Data Availability

The data that support the findings of this study are available from the corresponding author upon reasonable request.
